# Prostate bed irradiation with alternative radio-oncological approaches (PAROS) - a prospective, multicenter and randomized phase III trial

**DOI:** 10.1186/s13014-019-1325-x

**Published:** 2019-07-10

**Authors:** Stefan A. Koerber, Sonja Katayama, Anja Sander, Cornelia Jaekel, Matthias F. Haefner, Juergen Debus, Klaus Herfarth

**Affiliations:** 1 Department of Radiation Oncology, Heidelberg University Hospital, Heidelberg, Germany; 2 National Center for Tumor diseases (NCT), Heidelberg, Germany; 3 Heidelberg Institute of Radiation Oncology (HIRO), Heidelberg, Germany; 4 Institute of Medical Biometry and Informatics, Heidelberg University, Heidelberg, Germany; 5 Heidelberg Ion-Beam Therapy Center (HIT), Department of Radiation Oncology, Heidelberg University Hospital, Heidelberg, Germany; 6 German Cancer Consortium (DKTK), Partner site Heidelberg, Heidelberg, Germany; 7 Clinical Cooperation Unit Radiation Oncology, German Cancer Research Center (DKFZ), Heidelberg, Germany

**Keywords:** Prostate cancer, Prostate bed, Hypofractionation, Protons, Radiotherapy, Salvage

## Abstract

**Background:**

For patients with treatment-naïve carcinoma of the prostate, hypofractionated irradiation becomes more and more popular. Due to the low α/β value of prostate cancer, increased single dose leading to a shortened treatment period seems to be safe and feasible. However, reliable data is lacking for post-prostatectomy patients so far. Further, the role of proton therapy is still under debate. Two prospective phase II trials with both, hypofractionated photon and proton therapy, provided promising results.

**Methods/ design:**

The PAROS trial is a prospective, multicenter and randomized phase III trial for men with localized prostate carcinoma after surgery. Post-prostatectomy patients will be randomized to either normofractionated radiotherapy (nRT) with photons (70.0/ 2.0 Gy), or hypofractionated radiotherapy (hRT) with photons (57.0/ 3.0 Gy) or hRT with protons (57.0/ 3.0 Gy relative biological effectiveness [RBE]). Block randomization is stratified by Gleason Score (≤ 7 vs. > 7) and treatment indication (adjuvant vs. salvage). The trial is planned to enroll 897 patients. The primary objective is to show an improvement in the bowel-score according to EORTC QLQ-PR25 after proton therapy compared to photon irradiation (week 12 vs. baseline). Secondary aims are non-inferiority of hRT compared to nRT with regard to biochemical progression-free survival (bPFS), overall survival (OS), quality of life and toxicity.

**Discussion:**

The present study aims to evaluate the role of hypofractionated radiotherapy to the prostate bed with photons and protons leading to significant impact on future management of operated men with prostate cancer.

**Trial registration:**

Deutsches Register klinischer Studien: DRKS00015231; registered 27 September 2018.

## Background

For patients with non-metastatic prostate carcinoma, surgery or radiotherapy with or without hormonal therapy (HT) are curative treatment options. After prostatectomy, irradiation can be performed as adjuvant therapy or after prostate specific antigen (PSA) rise. While several larger studies reported on the oncological benefit for postoperative radiotherapy [1, 2], there is only one prospective phase III trial evaluating the role of dose-escalated salvage irradiation so far [3]. One multicenter, prospective phase II study from Germany is currently analyzing the role of moderately dose-escalated salvage radiotherapy in combination with local hyperthermia [4].

For some years now, hypofractionated irradiation becomes more and more common for patients with prostate cancer. Many trials observed excellent clinical outcome after moderate hypofractionation for patients undergoing definitive radiotherapy [5–8]. As one of the largest studies, the CHHiP trial evaluated 3216 men with localized prostate cancer. With a median follow-up of 62.4 months, hypofractionated radiotherapy (hRT) with 60 Gy in 20 fractions was not inferior compared to conventional fractionation. At 5 years, 90.6% in the 60 Gy group and 88.3% in the 74 Gy group were free of biochemical or clinical failure [5]. Nowadays, hypofractionation is frequently proclaimed as the “new standard of care” for definitive radiotherapy of patients with prostate cancer. However, in the postoperative setting reliable data is missing with regard to hypofractionation. Few studies with mostly small numbers of patients reported on feasibility and toxicity. When using moderate hRT, postoperative radiotherapy seems to be safe and provided promising clinical results [9–11]. Lewis et al. observed no acute grade 3 toxicity and a 4-year bPFS of 75% in a cohort of 56 men. All patients obtained image-guided intensity-modulated radiation therapy (IMRT) in 2.5 Gy fractions [12]. Our institution also tested different approaches for hypofractionation after surgery: The PRIAMOS 1 trial evaluated treatment safety and toxicity of hRT of the prostate bed using IMRT and daily image-guidance. In this prospective phase II trial, 40 men received adjuvant or salvage irradiation with single doses of 3.0 Gy up to a total dose of 54.0 Gy. Treatment was tolerated well with no recorded side effects grade 3+ [13]. Very similar results were obtained when using proton therapy instead of photons. With the use of protons, patient-reported bowel-score according to EORTC QLQ-PR25 questionnaire was already improved at week 10 and reached borderline significance when compared to photon therapy [*data unpublished*]. Therefore, larger and randomized trials are of great interest evaluating the role of hRT for patients after prostatectomy.

The PAROS trial is designed as a prospective, multicenter and randomized 3-arm phase III trial evaluating toxicity and efficacy of hypofractionation for prostate cancer patients undergoing adjuvant or salvage irradiation.

## Methods

### Primary and secondary endpoints

The primary endpoint is defined as the change in the bowel-score according to EORTC QLQ-PR25 from baseline to 12 weeks after start of proton therapy compared to photon irradiation.

Secondary endpoints are bPFS after 5 years, quality of life (QoL) according to EORTC QLQ-C30 and –PR25 after 2 and 5 years, clinical symptoms and toxicity according to National Cancer Institute Common Terminology Criteria for Adverse Effects (NCI CTCAE) version 5.0 after 2 and 5 years as well as overall survival (OS) after 5 years.

### Trial design

**Fig. 1:**
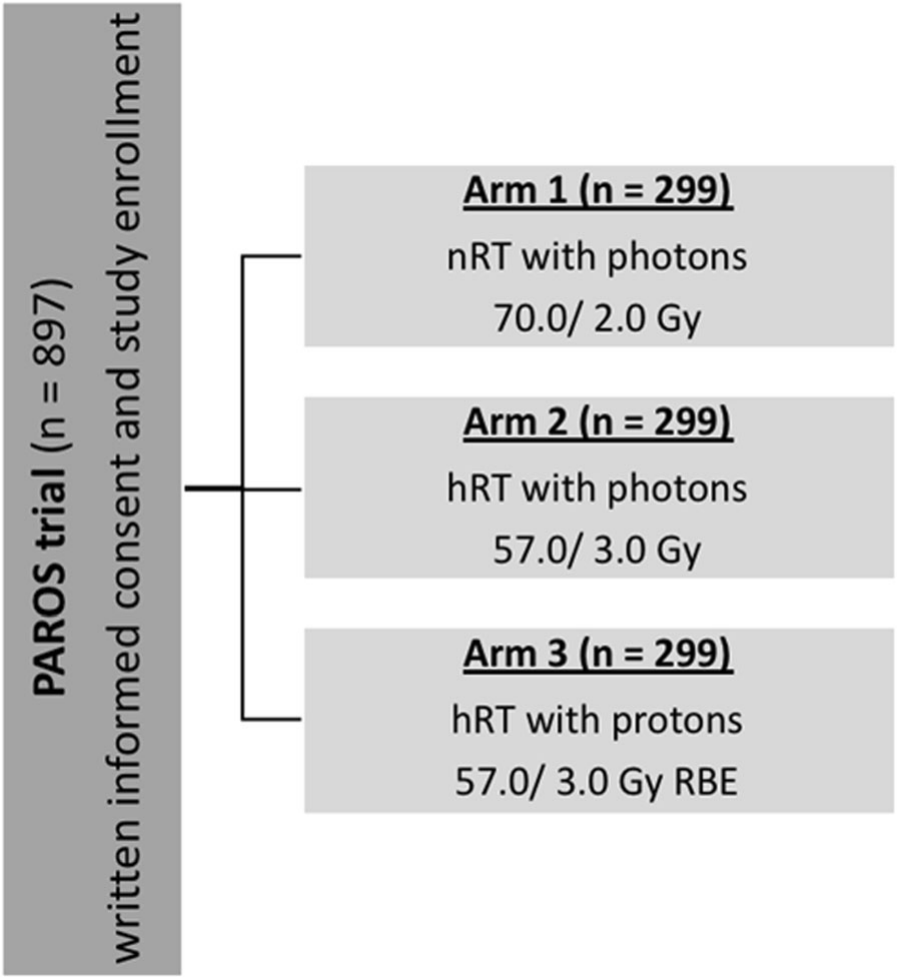
PAROS trial flowsheet. nRT = normofractionated radiotherapy; hRT = hypofractionated radiotherapy; Gy = Gray; RBE = relative biological effectiveness

The trial is a prospective, multicenter, randomized phase III trial of patients with operated prostate carcinoma and is planned to enroll 897 patients with localized prostate cancer after prostatectomy. Patients will be randomized to one of the three arms: nRT with photons, hRT with photons or hRT with protons. Total dose is 70.0 Gy in 35 fractions for nRT with photons (arm 1), 57.0 Gy in 19 fractions for hRT with photons (arm 2) and 57.0 Gy relative biological effectiveness (RBE) in 19 fractions for hRT with protons (arm 3), respectively (Fig. 1). The study was designed as a multicenter trial in at least seven radio oncological centers in Germany and is conducted in accordance with the Declaration of Helsinki and the guidelines of Good Clinical Practice in their current versions. Before trial initiation, the study was approved by the local institutional review board and the expert committee of the German Society of Radiation Oncology. Written informed consent will be obtained from all patients prior to inclusion into the trial.

### Inclusion and exclusion criteria

Inclusion criteria:

- histologically proven, localized prostate cancer with classification according to Gleason Score/ WHO grading and initial PSA value
- indication for adjuvant or salvage irradiation of the prostate bed after prostatectomy
- no evidence of nodal/ bone or organ metastases in imaging according to national guidelines for patients with PSA persistence/ relapse and a PSA value of > 1 ng/ml
- Karnofsky index ≥70%
- age ≥ 18 years
- written informed consent

Exclusion criteria:

- hormonal therapy
- macroscopic tumor/ R2 resection margin
- nodal metastases
- distant metastases (M1)
- previous pelvic irradiation
- concurrent participation in other clinical trials, which might influence the results of the present study
- active medical implants without treatment approval at the time of proton therapy (e.g. defibrillator, pacemaker)

### Pretreatment preparations/ randomization

When meeting the inclusion criteria, patients are informed about the trial including potential risks and benefits. After written informed consent, patients will be allocated (1:1:1) in concealed fashion into one of the three treatment arms (arms 1–3). Block randomization will be carried out with permuted block sizes and stratified by Gleason Score (GS) (≤ 7 vs. > 7) and treatment indication (adjuvant vs. salvage) using a centralized web-based tool (www.randomizer.at). Participating sites have to register at the randomization platform before. All required documentation will be transferred to the study center (Study Administration, Department of Radiation Oncology, Heidelberg University Hospital, INF 400, 69,120 Heidelberg).

### Irradiation

Treatment planning and application will be performed at the site of study enrollment. For centers without a proton unit, patients randomized in arm 3 will be referred to one participating site with protons available. Patient positioning and immobilization will be performed according to institutional standards. Photon irradiation will be applied in IMRT/ image-guided radiotherapy (IGRT) technique, proton beam therapy is performed as active beam application (raster scanning method).

For contouring, the bladder, rectum, posterior third of the rectum, bowel (small bowel, colon/ sigma) and femoral heads will be defined as organs-at-risk (OAR). Target volume delineation will be done according to Radiation Therapy Oncology Group (RTOG) guidelines (post-OP prostate cancer). The planning target volume (PTV) will be obtained by adding a 5 mm (anterior-posterior; superior-inferior) and 7 mm (lateral direction) margin to the clinical target volume (CTV), respectively. Total dose will be prescribed to 50% of the PTV for each treatment arm. Equivalent dose in 2.0 Gy/ fraction (ED2) for hRT (arm 2 + 3) is 69.7 Gy, calculated for 2.0 Gy single dose considering an α/β value of 2.5. For proton irradiation, dose will be calculated with an RBE of 1.1 according to ICRU 78.

Dose to OAR may not exceed the tolerance dose (TD) 5/5 (toxic dose leading to 5% severe complications over 5 years). The maximum dose of PTV_rectum (defined as the intersection between PTV and the rectum) is 70.0 Gy (arm 1) and 57.0 Gy (RBE) (arm 2 + 3), respectively. In the hRT arm, maximal EQD2 for the rectum is 66.5 Gy (RBE) (α/β value of 4).

### Follow-up/ evaluation of efficacy and safety parameters

The first follow-up examination will be performed at 12 weeks after the start of irradiation. While regular urooncological aftercare will be organized according to national guidelines, follow-up vists within the trial will be scheduled after 6, 12, 24, 36 and 60 months (Table 1). PSA levels can also be received from the treating urologist every 3months for the first 2 years and semi-annually thereafter.

**Table 1:**
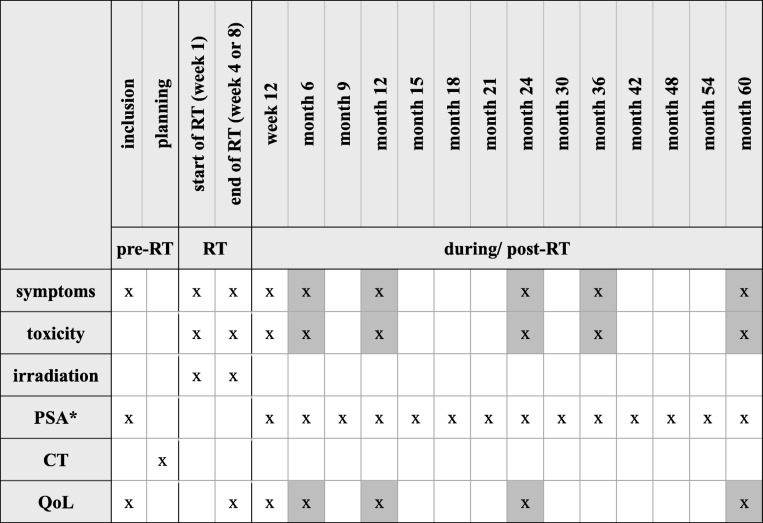
Time schedule for the present trial

*RT* radiotherapy, *PSA* prostate specific antigen, *CT* computed tomography, *QoL* quality of life

*obtained from treating urologist or participating radio oncological center

grey = postal query or query by phone is permitted

Acute and chronic toxicity will be evaluated according to NCI CTCAE version 5.0 during and after the treatment. EORTC QLQ-C30 and -PR25 questionnaires are used to collect data on QoL.

Biochemical failure is defined as two consecutive increases of PSA from nadir according to national guidelines [14–17]. bPFS is defined as time from first diagnosis to biochemical failure. OS is defined as time from the first diagnosis to death from any course. If the respective event has been observed, the patient is censored at the date of the last follow-up examination.

### Sample size calculation

The sample size calculation is based on the primary endpoint change in the bowel-score according to EORTC QLQ-PR25 from baseline to 12 weeks. Based on numerous trials, we assume a change of 6.5 points in the standard arm (arm 1). Based on the two phase II trials mentioned above, we assume a change of 6.5 points in arm 2 as well and a change of 2.5 points in arm 3. Equal standard deviation of 15 points is expected for all arms. In the primary analysis, arm 1 will be compared with arm 3 and arm 2 with arm 3. To control the overall type I error of 5%, local significance levels of 2.5% according to Bonferroni were applied in the sample size calculation. To achieve a power of 80% with the assumptions above, a sample size of 269 per arm results for the two-sided t-test. Calculations have been performed using PASS 14.0.8. The primary analysis will be performed applying the Bonferroni Holm procedure and the factors used in the stratified randomization will be considered. This will lead to an increase in power. Based on the experiences from previous studies, a conservative rate of drop-outs and loss to follow-ups of 10% is expected. To compensate for this loss in information, 299 patients per arm are required resulting in a total of 897 patients to be randomized.

### Statistical analysis

In the primary analysis, arm 1 vs. arm 3 and arm 2 vs. arm 3 will be compared regarding the primary endpoint applying a linear regression model including the stratification variables used in the randomisation procedure and center. Primarily, the analysis will be based on the Intention to treat population which consists of all randomized patients treated at least for 1 week in the arm as randomized. Missing values for the primary endpoint will be imputed using multiple imputations under the “missing at random” assumption and the results will be pooled. To control the global type I error rate with 5%, the Bonferroni Holm procedure will be applied. In case that at least one of the two comparisons can be considered significant, arm 1 vs. 2 will be compared at a significance level of 5%, still controlling the global significance level of 5%.

In addition, a complete case analysis will be carried out. In the per protocol set only patients who were treated as described in the protocol with complete documentation of relevant data will be considered. This set will be analyzed as well as sensitivity analysis.

Time to event endpoints will be analyzed using Cox proportional hazard models adjusting for the stratification variables used in the randomization procedure. All secondary endpoints will be descriptively analyzed using statistical methods as appropriate for the underlying distribution of the data.

Safety endpoints will be analyzed based on the safety population which comprises all patients who had at least 1 day of treatment, considered in the arm as treated. Rates of acute and chronic toxicity will be calculated together with corresponding 95 confidence intervals for group comparisons. Descriptive *p*-values for the chi-squared test will be provided. Statistical methods will be used to assess the quality of the data, homogeneity of treatment groups, endpoints and safety of the two intervention groups. Details of the statistical analysis will be fixed at the latest in the Statistical Analysis Plan (SAP) to be prepared before database closure. This also includes the definition of the analysis populations.

## Discussion

After prostatectomy, irradiation is recommended for several patients with prostate cancer due to various criteria like positive resection margins or PSA relapse. Although prostate specific membrane antigen (PSMA) positron emission tomography (PET)/CT is now available for recurrent disease, detection rates are limited; especially for low and very low PSA levels. Several studies reported on a probability of about 50% in detecting macroscopic tumor at PSA levels of < 0.5 ng/ml [18, 19]. Currently, there is no evidence for prolonging adjuvant or salvage radiotherapy to reach higher detection rates from PSMA PET/CT. Therefore, radiotherapy is often prescribed to the prostate bed assuming this localization has the highest risk for prostate cancer cells.

Today, nRT with photons is considered as the standard of care for patients undergoing irradiation after prostatectomy. However, treatment time covers a period of several weeks for each patient so far. Due to the low α/β value of prostate cancer compared to relevant normal tissue, hypofractionation seems to be feasible and safe without an increase of late reactions. However, inconsistent data exist so far with regard to chronic side effects: While several studies reported on similar late toxicity when using postoperative, hRT [12, 20], Cozzarini et al. observed a higher risk of late urinary toxicity in a relatively small group of patients who underwent hypofractionation. In this retrospective analysis, the 5-year risk of severe (grade ≥ 3) late urinary side effects increased in the heterogeneous cohort of patients treated with different hRT regimes compared to men treated with normofractionation [21]. Even though results should be interpreted with caution due to the retrospective character of the study and the small number of patients in the different hRT subgroups, additional research is imperative.

Further, the role of post-prostatectomy proton therapy is still unclear. Due to its physical characteristics, dose to the target volume can be achieved more efficiently with the use of particles like protons while leading to an optimized sparing of the surrounding OAR. Apart from previous in-house data, normofractionated proton therapy is likely to be feasible and safe with a favorable toxicity profile also in postoperative setting. Deville et al. observed no acute and late adverse effects grade ≥ 3 in a cohort of 100 men undergoing post-prostatectomy nRT with a median follow-up of 25 months [22]. Nevertheless, the potential reduction of side effects by the use of protons is highly debated due to the fact, that parts of the OAR are included in the target volume for both, protons and photons. Prospective, randomized trials are lacking so far.

The PAROS trial aims to evaluate the role of hypofractionated irradiation with either photons or protons for patients with prostate carcinoma after surgery. The study results will impact future management and treatment recommendations for this large group of patients.

## Trial status/ planned end of the study

Recruitment of the present trial was initiated in late 2018 and is expected to end in Q4 of 2023. The regular end of study participation for each patient is 60 months after the end of treatment (last patient out expected in Q4 2028/ Q1 2029).

## Data Availability

The datasets used and/or analysed during the current study are available from the corresponding author on reasonable request.

## Abbreviations

bPFS: biochemical progression-free survival
CT: Computed tomography
CTV: Clinical target volume
ED2: Equivalent dose in 2.0 Gy/ fraction
GS: Gleason Score
Gy: Gray
hRT: hypofractionated radiotherapy
HT: Hormonal therapy
IGRT: Image-guided radiotherapy
IMRT: Intensity-modulated radiation therapy
NCI CTCAE: National Cancer Institute Common Terminology Criteria for Adverse Effects
nRT: normofractionated radiotherapy
OAR: Organs at risk
OS: Overall survival
PET: Positron emission tomography
PSA: Prostate specific antigen
PSMA: Prostate specific membrane antigen
PTV: Planning target volume
QoL: Quality of Life
QoL: Quality of life
RBE: Relative biological effectiveness
RTOG: Radiation Therapy Oncology Group
TD: Tolerance dose
WHO: World health organization
